# Supplementary material for: Mercury Chloride but Not Lead Acetate Causes Apoptotic Cell Death in Human Lung Fibroblast MRC5 Cells via Regulation of Cell Cycle Progression
Source: Int J Mol Sci. 2021 Mar 2;22(5):2494. doi: 10.3390/ijms22052494 (PMC7958599; doi:10.3390/ijms22052494)
Supplement: Supplementary file 1 [file ijms-22-02494-s001.pdf]

International Journal of
Molecular Sciences

MDPI

Supplementary Material

Mercury Chloride but not Lead Acetate Causes Apoptotic Cell Death in Human Lung Fibroblast MRC5 Cells via Regulation of Cell Cycle Progression

Ji-Young Kim[1], Mi-Jin An[1], Geun-Seup Shin[1], Hyun-Min Lee[1], Mi Jin Kim[1], Chul-Hong Kim[1], and Jung-Woong Kim[1],*

[1] Department of Life Science, Chung-Ang University, Seoul 06974, South Korea;
* Correspondence: jungkim@cau.ac.kr; Tel.: +82-2-820-6682, Fax: +82-2-815-6682

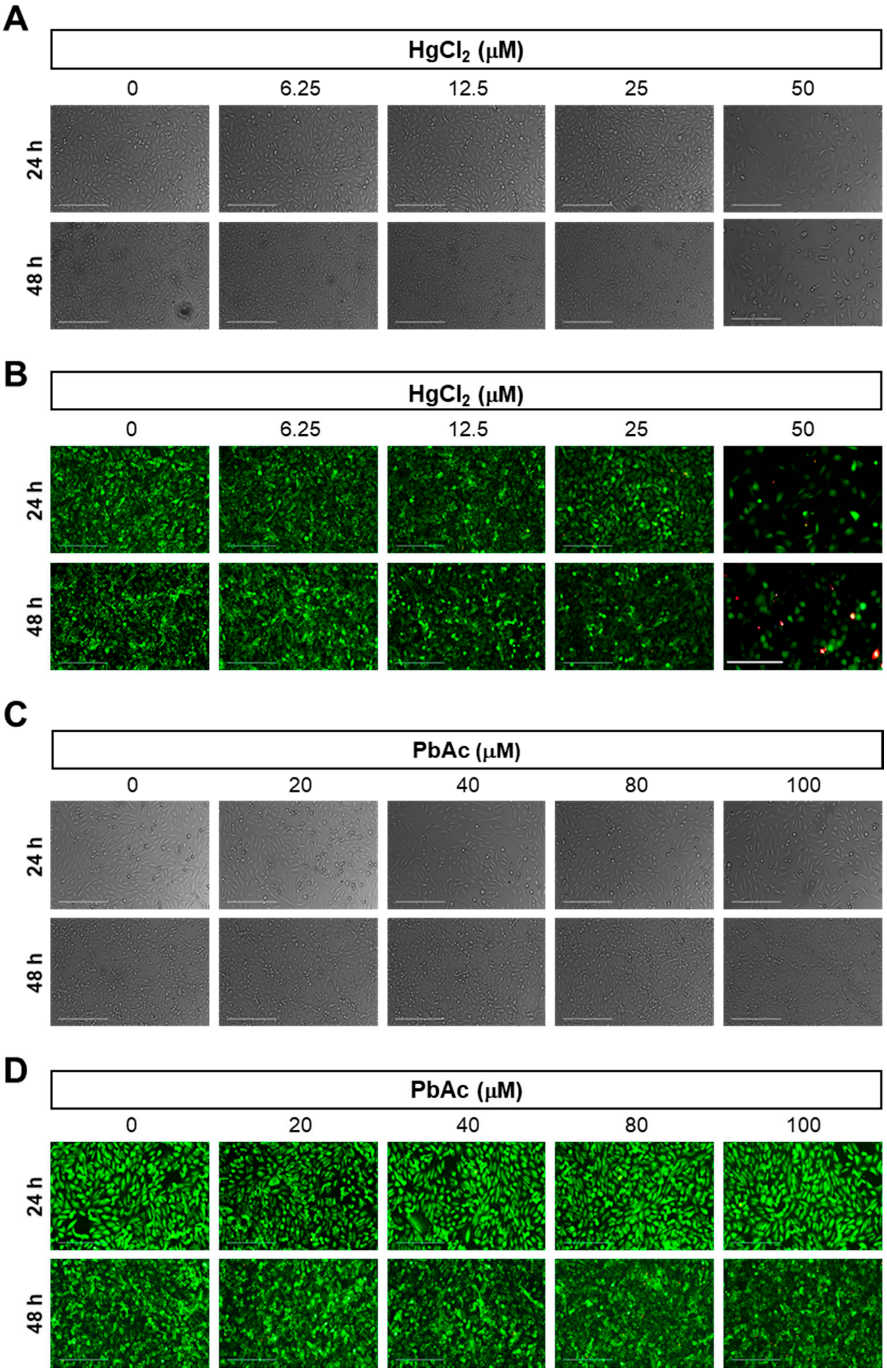

Figure S1. Effect of heavy metals on morphology of MRC5 cells. (**A and C**) The morphology of MRC5 cells was observed by phase-contrast microscopy after treatment with indicated concentration of $HgCl_2$ and PbAc for 24 or 48 h. (**B and D**) MRC5 cells were stained with calcein-AM (green) and ethidium homodimer (red) by the live/dead assay. EtOH were used as the negative control. Images are representative of three independent experiments. Scale bars represent 200 μm.

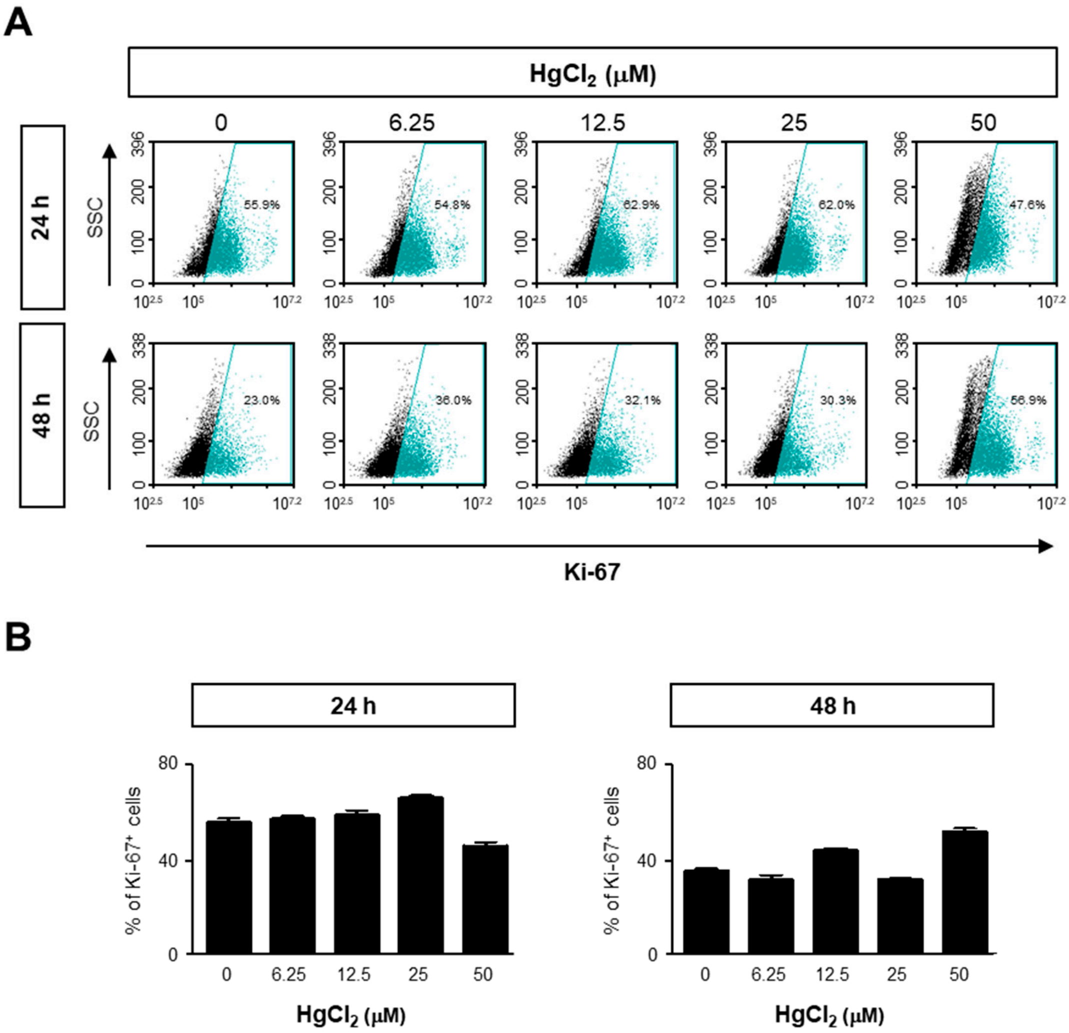

Figure S2. $HgCl_2$ treatment reduced the proliferation of MRC5 cells. (**A**) The heavy metals-treated MRC5 cells were immunostained with anti-Ki-67 antibodies. The cells were counted by FACS analysis. (**B**) The percentages of Ki-67-positive population are represented as the mean ± S.E.M. of three independent experiments (n = 6), each performed in triplicate.

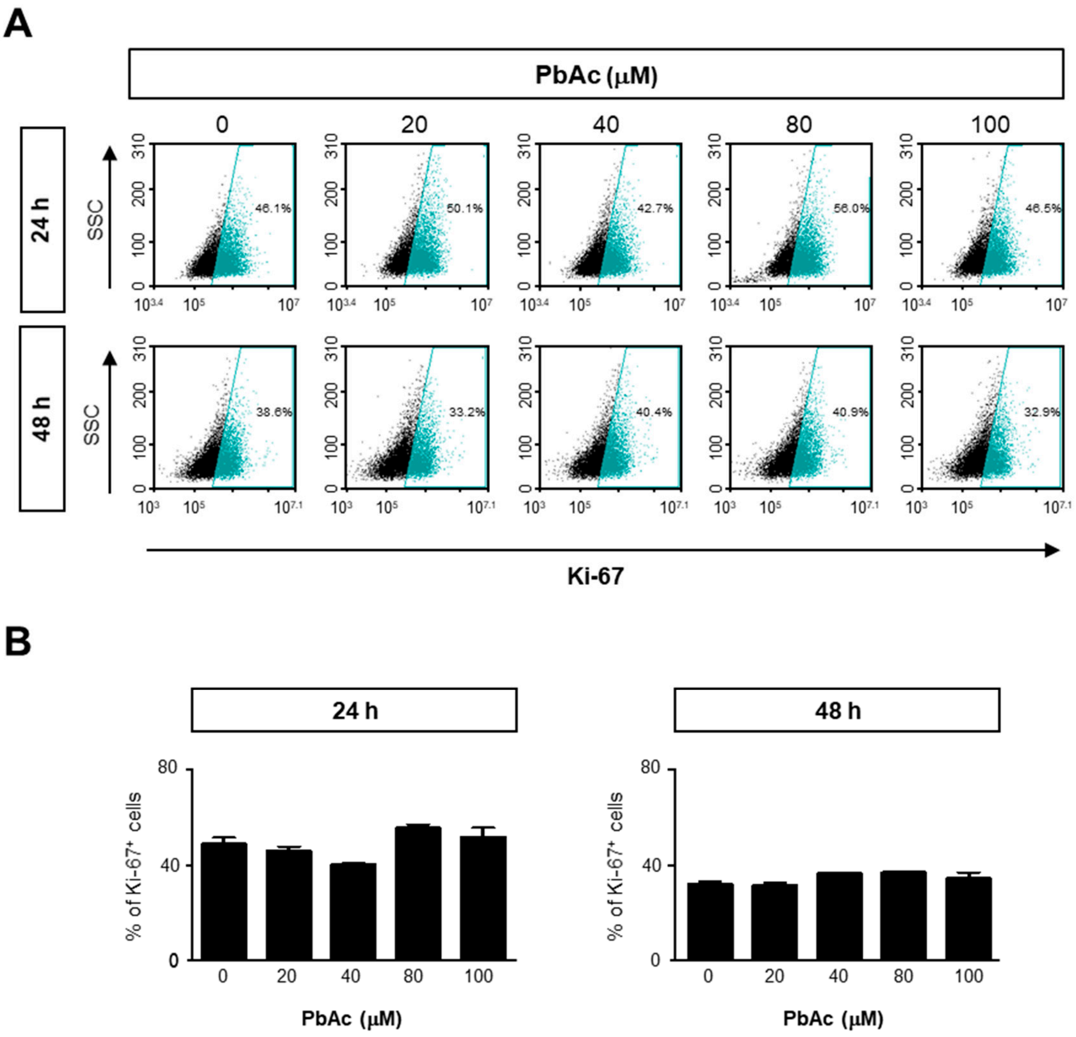

Figure S3. PbAc treatment reduced the proliferation of MRC5 cells. (**A**) The heavy metals-treated MRC5 cells were immunostained with anti-Ki-67 antibodies. The cells were counted by FACS analysis. (**B**) The percentages of Ki-67-positive population are represented as the mean ± S.E.M. of three independent experiments (n = 6), each performed in triplicate.

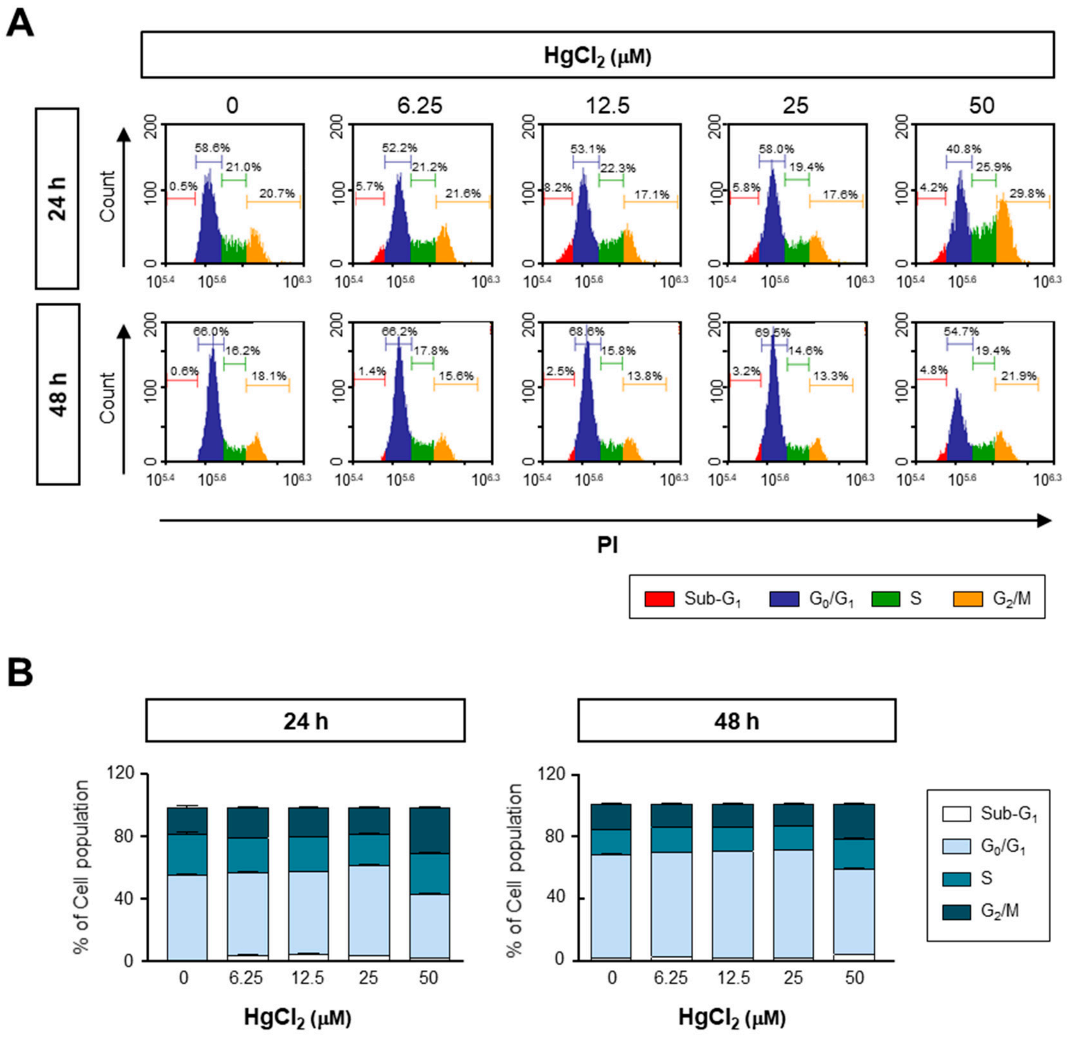

Figure S4. Cell cycle progression in $HgCl_2$ treatment in MRC5 cells. (**A**) The heavy metals-treated MRC5 cells were stained with propidium iodide (PI), and the cell cycle was measured using FACS analysis. (**B**) The percentages of population in the sub-G_1, G_0/G_1, S, and G_2/M-phases are represented as the mean ± S.E.M. of three independent experiments (n = 6), each performed in triplicate.

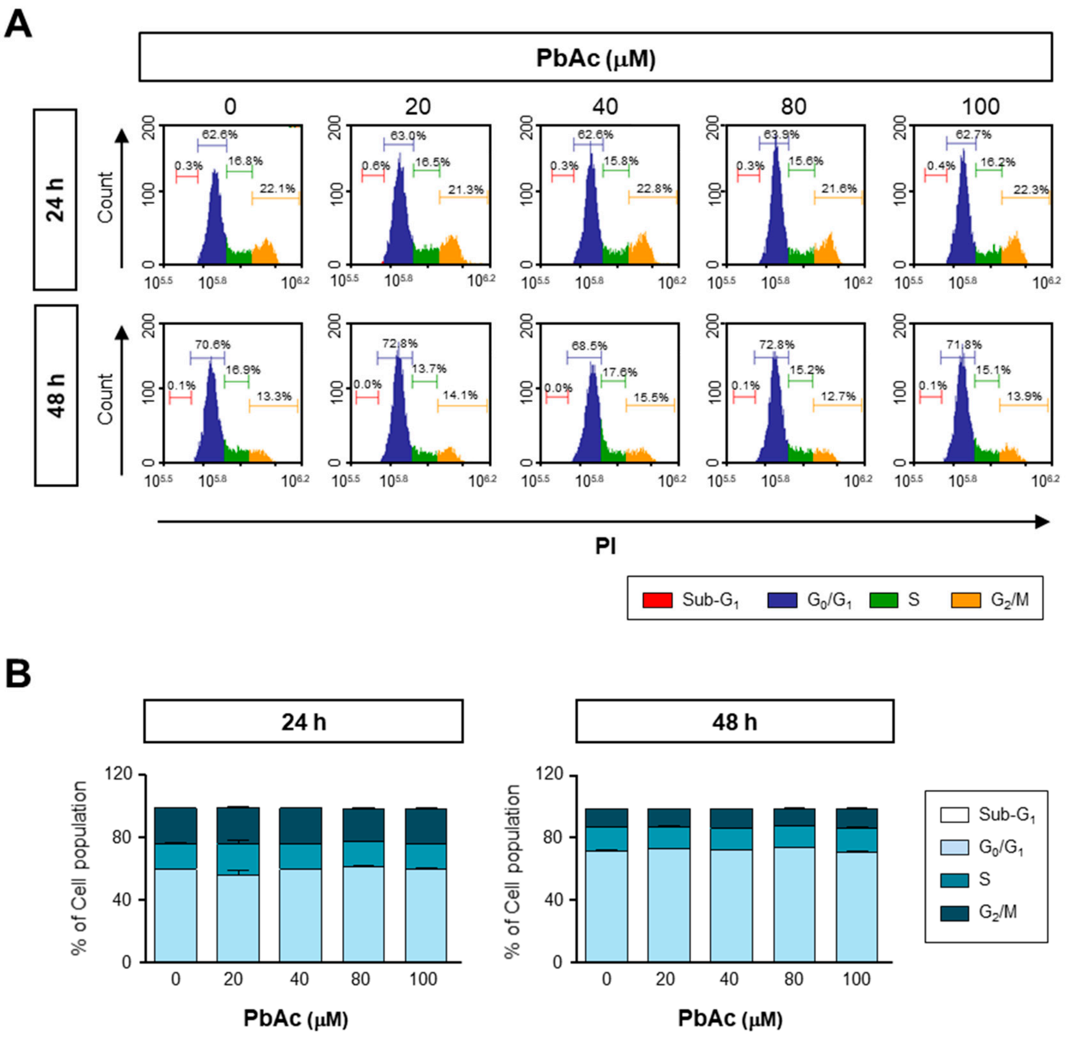

Figure S5. Cell cycle progression in PbAc treatment in MRC5 cells. (**A**) The heavy metals-treated MRC5 cells were stained with propidium iodide (PI), and the cell cycle was measured using FACS analysis. (**B**) The percentages of population in the sub-G_1, G_0/G_1, S, and G_2/M-phases are represented as the mean ± S.E.M. of three independent experiments (n = 6), each performed in triplicate.

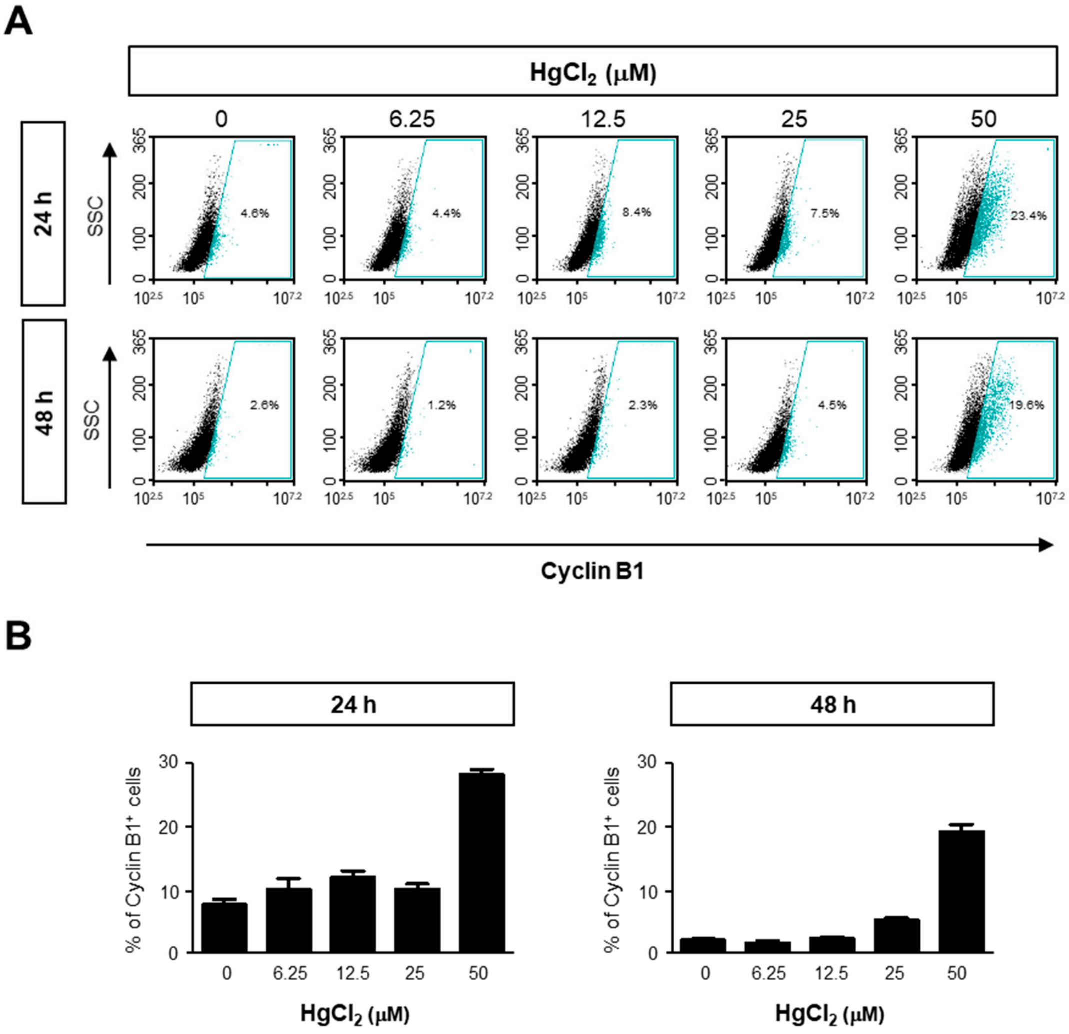

Figure S6. The effect of HgCl2 on cyclin B1 expression in MRC5 cells. (**A**) Cells were treated with indicated concentration of heavy metals for 24 or 48 h. Cells were fixed with 1 % PFA and stained with anti-cyclin B1 antibody. The cyclin B1 expression were analyzed by FACS analysis. (**B**) The percentages of cyclin B1-positive cells are represented as the mean ± S.E.M. of three independent experiments (n = 6), each performed in triplicate. EtOH was used as the negative control.

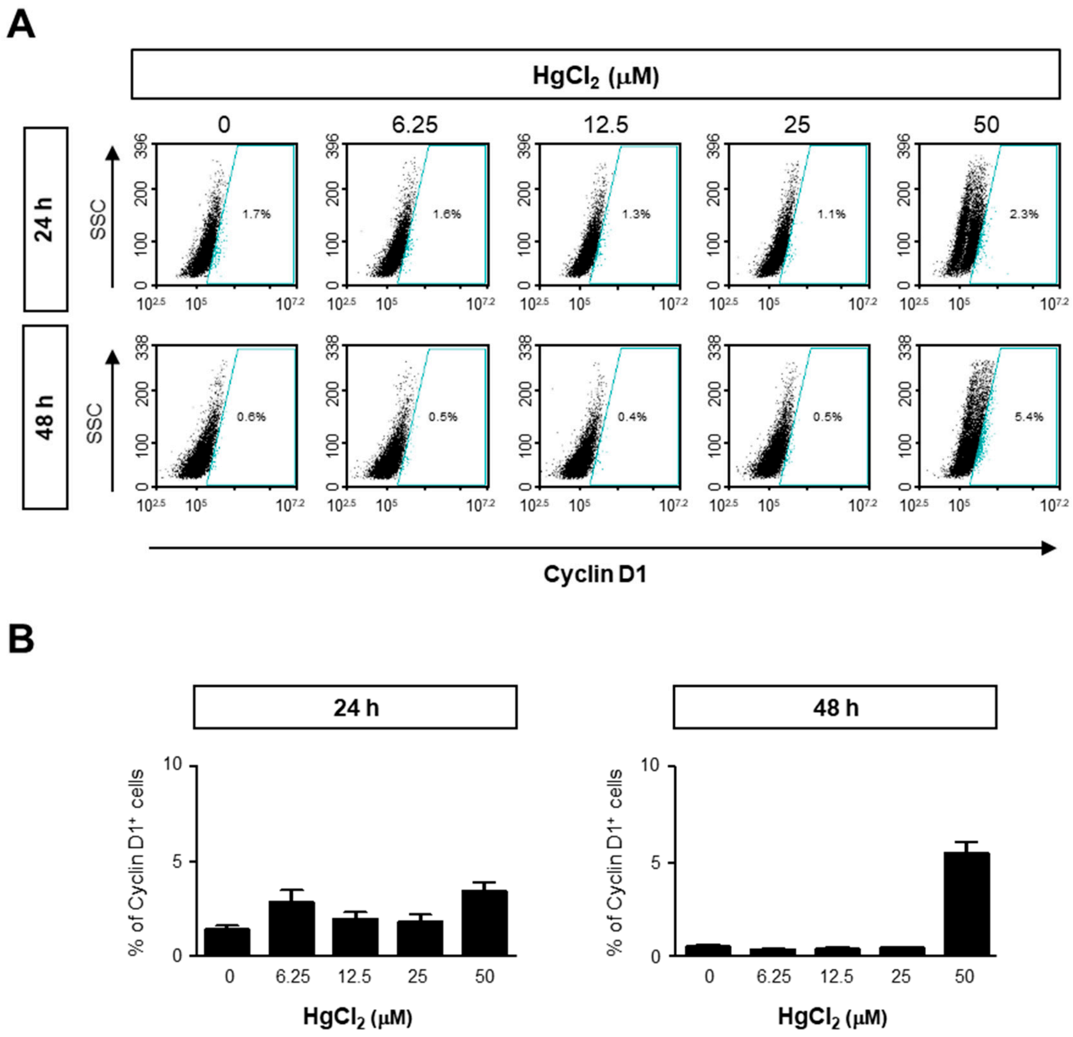

Figure S7. The effect of $HgCl_2$ on cyclin D1 expression in MRC5 cells. (**A**) Cells were treated with indicated concentration of heavy metals for 24 or 48 h. Cells were fixed with 1 % PFA and stained with anti-cyclin D1 antibody. The cyclin D1 expression were analyzed by FACS analysis. (**B**) The percentages of cyclin D1-positive cells are represented as the mean ± S.E.M. of three independent experiments (n = 6), each performed in triplicate. EtOH was used as the negative control.

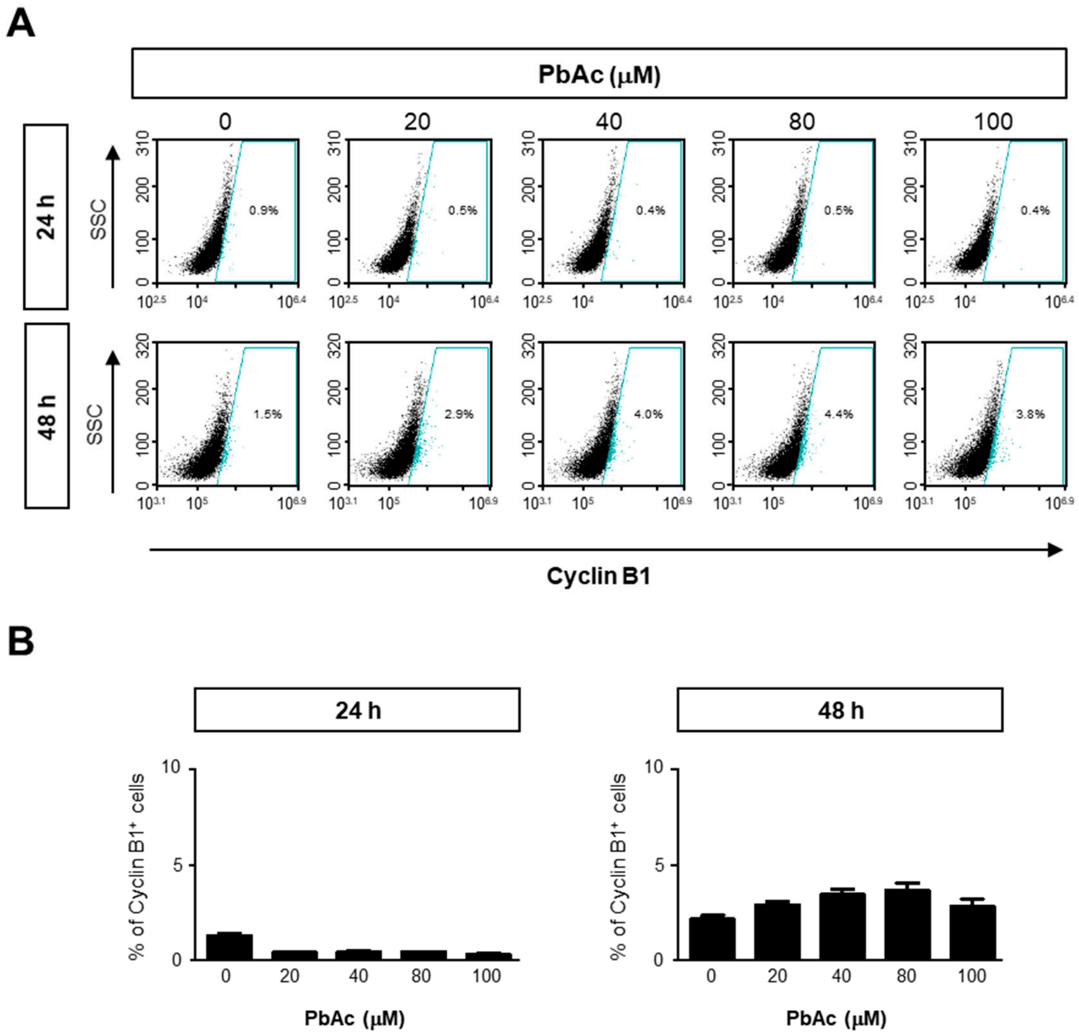

Figure S8. The effect of PbAc on cyclin B1 expression in MRC5 cells. (**A**) Cells were treated with indicated concentration of heavy metals for 24 or 48 h. Cells were fixed with 1 % PFA and stained with anti-cyclin B1 antibody. The cyclin B1 expression were analyzed by FACS analysis. (**B**) The percentages of cyclin B1-positive cells are represented as the mean ± S.E.M. of three independent experiments (n = 6), each performed in triplicate. EtOH was used as the negative control.

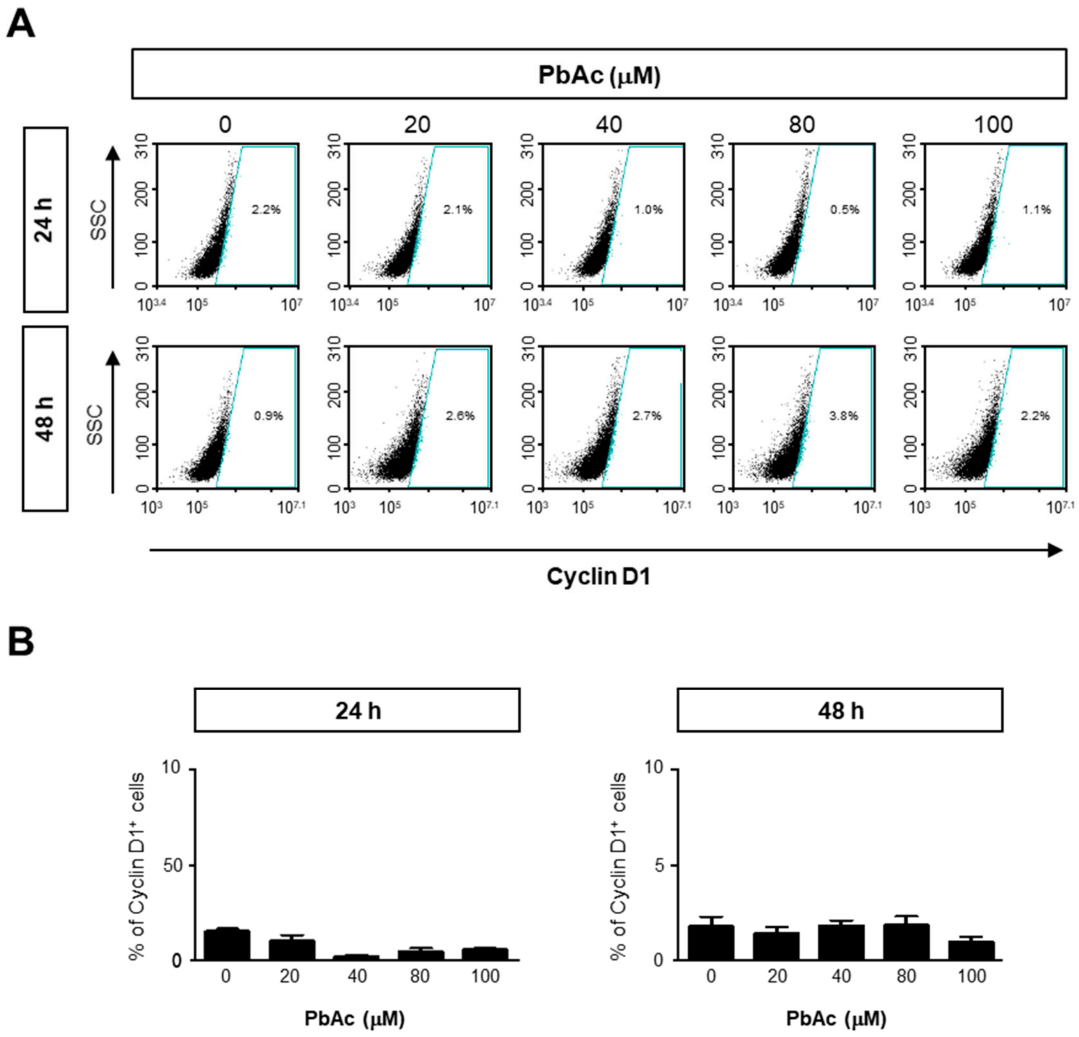

Figure S9. The effect of PbAc on cyclin D1 expression in MRC5 cells. (**A**) Cells were treated with indicated concentration of heavy metals for 24 or 48 h. Cells were fixed with 1 % PFA and stained with anti-cyclin D1 antibody. The cyclin D1 expression were analyzed by FACS analysis. (**B**) The percentages of cyclin D1-positive cells are represented as the mean ± S.E.M. of three independent experiments (n = 6), each performed in triplicate. EtOH was used as the negative control.

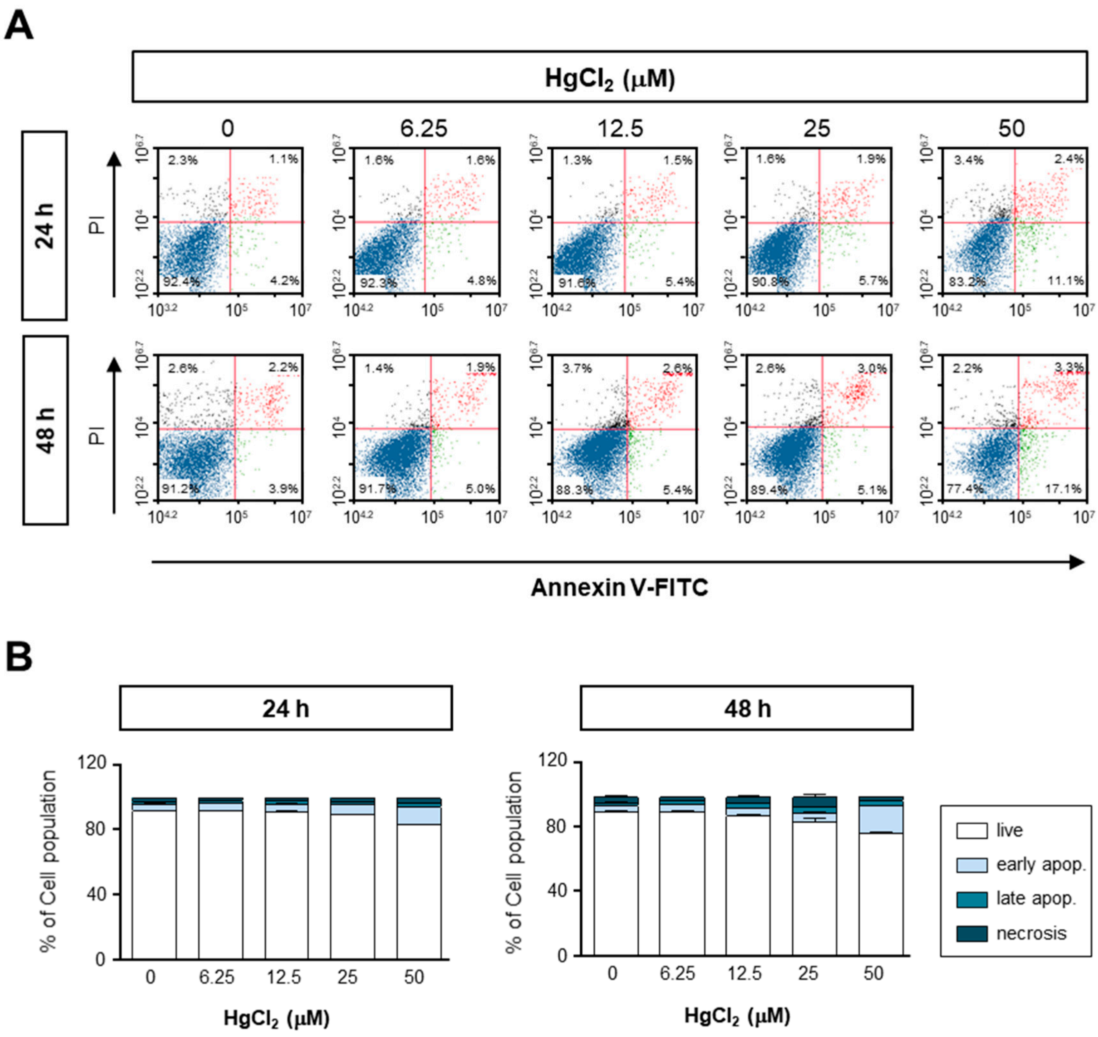

Figure S10. Effect of apoptotic cell death upon $HgCl_2$ treatment in MRC5 cells. (**A**) Cells were treated with indicated concentration of heavy metals for 24 or 48 h, and were double-stained with annexin V-FITC and PI. The proportion of apoptotic cells was assessed by FACS analysis. The scatter plots represent PI (Y-axis) and annexin V-FITC (X-axis). (**B**) The percentages of cells in the live, early- and late-apoptotic, and necrotic stages are expressed as the mean ± S.E.M. of three independent experiments (n = 6), each performed in triplicate.

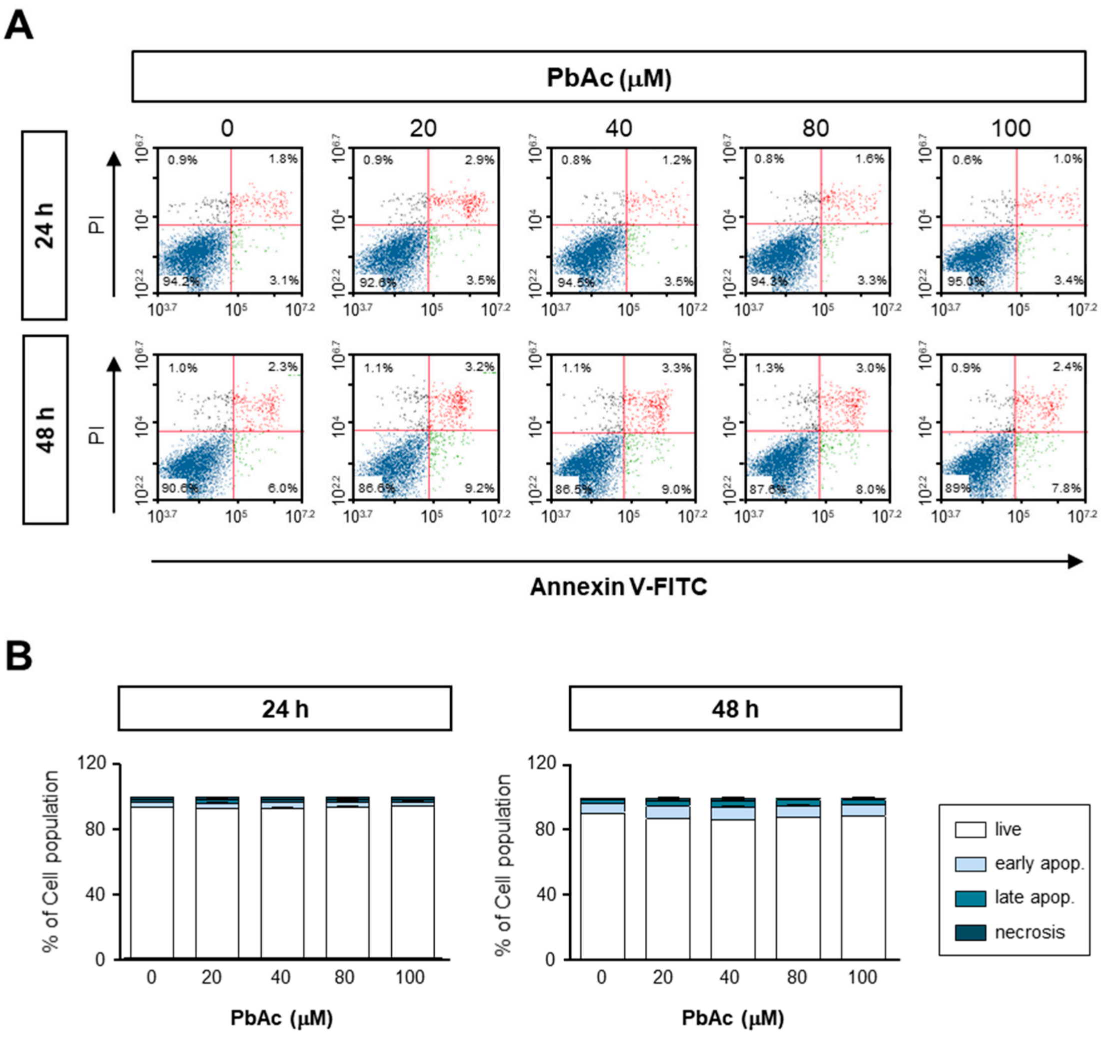

Figure S11. Effect of apoptotic cell death upon PbAc treatment in MRC5 cells. (**A**) Cells were treated with indicated concentration of heavy metals for 24 or 48 h, and were double-stained with annexin V-FITC and PI. The proportion of apoptotic cells was assessed by FACS analysis. The scatter plots represent PI (Y-axis) and annexin V-FITC (X-axis). (**B**) The percentages of cells in the live, early- and late-apoptotic, and necrotic stages are expressed as the mean ± S.E.M. of three independent experiments (n = 6), each performed in triplicate.

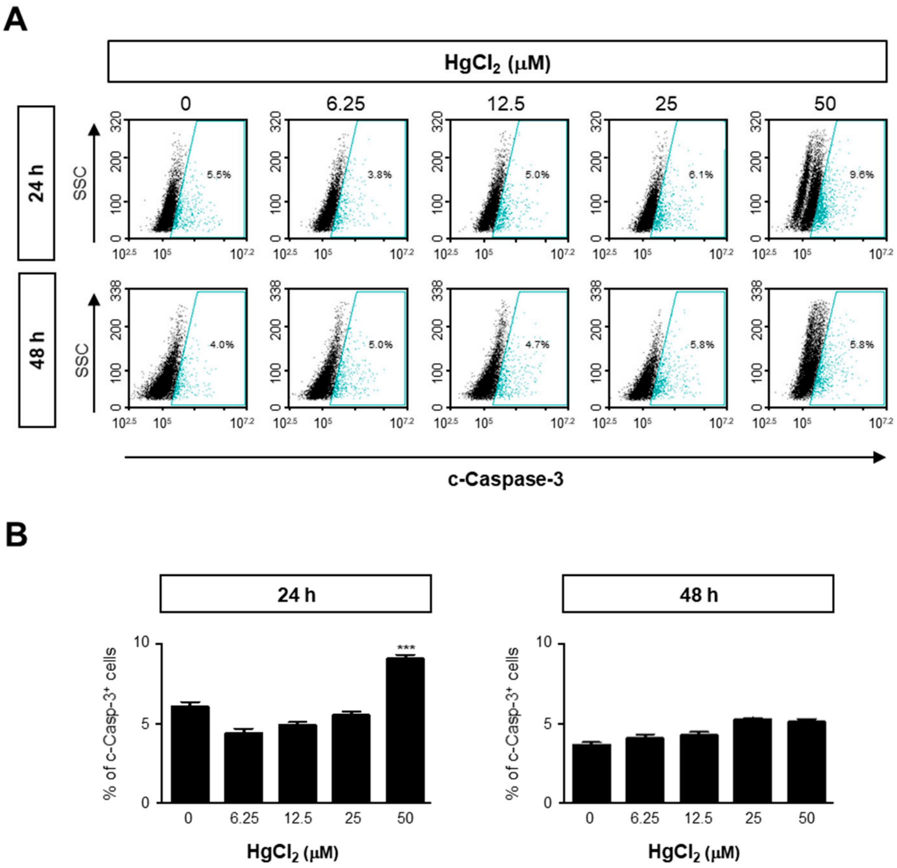

Figure S12. The effect of caspase-3 activity in $HgCl_2$-treated MRC5 cells. (**A**) Cells were treated with indicated concentration of heavy metals for 24 or 48 h. Cells were fixed with 1 % PFA and stained with anti-cleaved caspase-3 antibody. Expression levels of cleaved caspase-3 were analyzed by FACS analysis. (**B**) The percentages of cleaved caspase-3-positive cells are represented as the mean ± S.E.M. of three independent experiments (n = 6), each performed in triplicate. EtOH was used as the negative control.

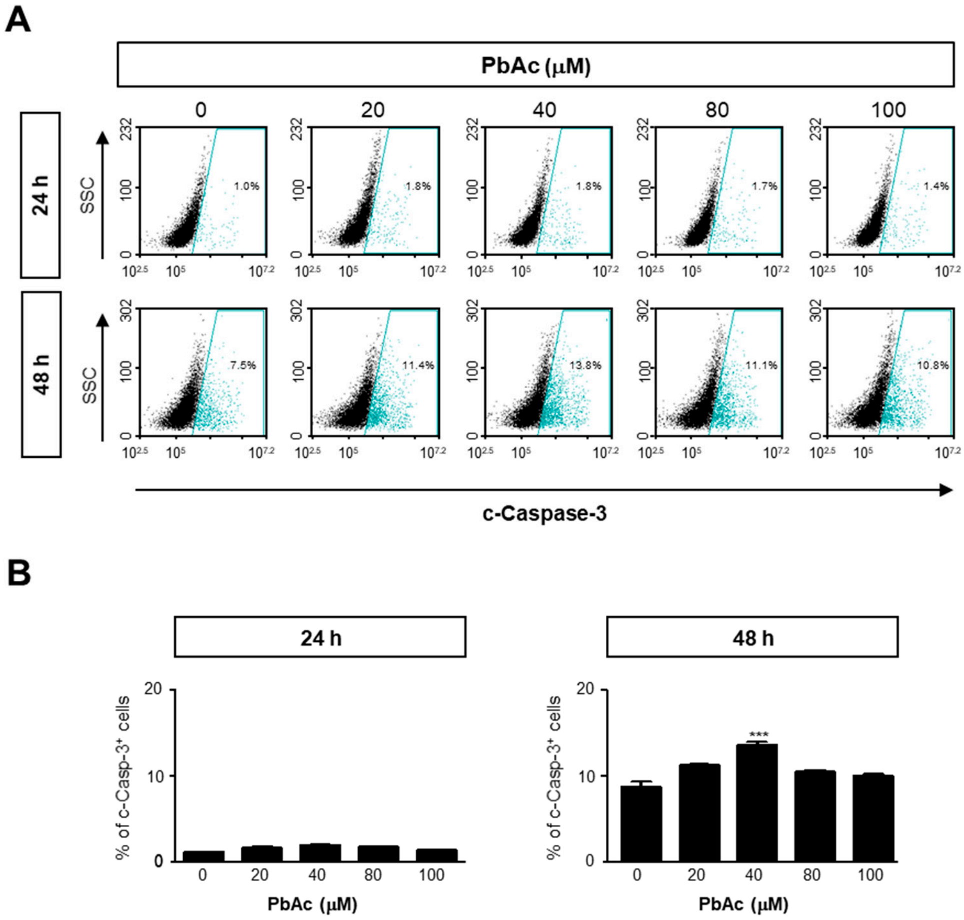

Figure S13. The effect of caspase-3 activity in PbAc-treated MRC5 cells. (**A**) Cells were treated with indicated concentration of heavy metals for 24 or 48 h. Cells were fixed with 1 % PFA and stained with anti-cleaved caspase-3 antibody. Expression levels of cleaved caspase-3 were analyzed by FACS analysis. (**B**) The percentages of cleaved caspase-3-positive cells are represented as the mean ± S.E.M. of three independent experiments (n = 6), each performed in triplicate. EtOH was used as the negative control.

Publisher's Note: MDPI stays neutral with regard to jurisdictional claims in published maps and institutional affiliations.

© 2021 by the authors. Submitted for possible open access publication under the terms and conditions of the Creative Commons Attribution (CC BY) license (http://creativecommons.org/licenses/by/4.0/).
